# Glyphosate-based herbicide exposure triggers genetic adaptation but not diversity collapse within bacterioplankton species

**DOI:** 10.1093/ismeco/ycag201

**Published:** 2026-08-18

**Authors:** Emma Derrick, Naíla Barbosa da Costa, Rowan D H Barrett, B Jesse Shapiro

**Affiliations:** Department of Microbiology and Immunology, McGill University, Montréal H3A 2B4, Canada; Institut national de la recherche scientifique - Centre Eau Terre Environnement (INRS-ETE), Québec G1K 9A9, Canada; Groupe de Recherche Interuniversitaire en Limnologie (GRIL), Département de sciences biologiques, Université de Montréal, Montréal H3C 3J7, Canada; Département Science et Technologie, Université Téluq, Québec G1K 9H6, Canada; Department of Biology, McGill University, Montréal H3A 1B1, Canada; Quebec Centre for Biodiversity Science, Montréal H3A 1B1, Canada; Department of Microbiology and Immunology, McGill University, Montréal H3A 2B4, Canada; Groupe de Recherche Interuniversitaire en Limnologie (GRIL), Département de sciences biologiques, Université de Montréal, Montréal H3C 3J7, Canada; Quebec Centre for Biodiversity Science, Montréal H3A 1B1, Canada; McGill Genome Centre, McGill University, Montréal H3A 0G1, Canada; McGill Centre for Microbiome Research, Montréal H4A 3J1, Canada

**Keywords:** experimental evolution, eco-evolutionary dynamics, selection, adaptation, freshwater bacteria, herbicides, metagenomics

## Abstract

Bacterial populations evolve rapidly in the lab when faced with experimentally applied selective pressures. Yet how bacteria evolve in nature, in more complex multi-species communities, is both challenging to study and essential to our understanding of ecosystem responses to rapid anthropogenic change. To track bacterial evolution in a semi-natural context, we applied Roundup, a glyphosate-based herbicide (GBH) that interferes with aromatic amino acid synthesis, as a selective pressure to 1000 L ponds containing bacterioplankton communities from a pristine lake. We show that both ecological and evolutionary changes can occur on short timescales after a strong selective pressure. Using metagenome-assembled genomes as a proxy for species, we found that GBH treatment substantially affected community diversity but did not purge within-species genetic diversity over the 4 weeks of the experiment. We identified several functional categories of genes consistently targeted by GBH selection across seven different species of bacteria. Genes involved in amino acid transport and metabolism were more likely to experience GBH-driven changes in allele frequency, including the gene *aroA* targeted by glyphosate, along with other potentially novel targets of selection. Together, these results show how environmental change can rapidly affect bacterial community structure and select for specific genetic targets without purging genetic diversity genome-wide.

## Introduction

Microbial communities and the populations within them form the foundation of all ecosystems on Earth [1]. Many studies have detailed how natural microbial communities change on an ecological level, with species changing in abundance in response to perturbations from environmental stressors [2–6]. It is becoming increasingly evident that microbial populations within communities can also evolve on the same time scales as ecological changes [7–9]. Rapid evolutionary change, for example, of pathogens within infected patients, can have consequences for virulence and disease persistence [10–13]. Evolutionary and ecological changes may occur independently [7] or may interact: for example, evolutionary diversification of a focal species can impact relative abundances of other species within the community [14]. Conversely, higher community diversity may promote or constrain the diversification of species within a community [8, 15, 16]. In all cases, ecological and evolutionary changes within microbial communities, along with their interactions, are expected to affect ecosystems’ functions.

What types of evolutionary changes can occur within a community? Under the stable ecotype model, selection on adaptive mutations can result in a genome-wide selective sweep, in which a genome with an adaptive allele expands clonally and purges genetic diversity from the population [17, 18]. Alternatively, if recombination is relatively frequent, an adaptive allele can be exchanged by horizontal gene transfer and spread through the population in a gene-specific sweep, purging diversity in one part of the genome while maintaining genome-wide diversity [19, 20]. Or, if an adaptive allele is originally present in the population on multiple genetic backgrounds, then multiple lineages carrying the adaptive allele may increase in frequency resulting in a soft sweep [21]. Both soft and gene-specific selective sweeps allow adaption to proceed without a collapse of genome-wide diversity.

Studies of natural bacterial populations in freshwater lakes have identified evolutionary patterns consistent with sweeps, but it remains unclear if these were driven by selection or genetic drift. Bendall *et al*. [22] observed a gradual loss of diversity over 9 years in one population of bacteria, consistent with a genome-wide sweep, as well as other populations with low diversity in small genomic regions, consistent with past gene-specific sweeps. More recently, Rohwer *et al*. [23] quantified changes in species abundance and diversity throughout a 20-year time series and identified a possible soft sweep in *Nanopelagicus*. While these studies suggest that selective sweeps are occurring in natural bacterioplankton populations, neither could attribute sweeps to a known selective pressure. Another recent study applied a known selective pressure (liming) to natural bacterial tree hole communities but failed to detect an evolutionary response to selection and only a weak ecological (species sorting) response, likely due to high community variability among tree holes [24].

There is growing concern about pollution from the runoff of agrochemicals such as glyphosate-based herbicides (GBHs) on aquatic communities [25]. Regulations in Canada limit the concentration of glyphosate permitted for chronic (<800 μg/L) and acute (<27 000 μg/L) aquatic contamination [26]. However, these guidelines are based on toxicity to eukaryotes and ignore potential effects on bacteria. Previous studies have shown that GBH alters the composition of bacterial communities in water [2] and honeybee microbiomes [5]. Further, GBH can cross-select for antibiotic resistance genes in soil [27] and aquatic [28] communities. GBH inhibits plant growth by interfering with the shikimate pathway and preventing downstream synthesis of essential aromatic amino acids [29]. Specifically, glyphosate competitively binds 5-enolpyruvylshikimate 3-phosphate synthase (EPSPS), encoded by the *aroA* gene [30]. Bacteria with different EPSPS alleles encoding specific amino acid changes vary in their resistance to glyphosate, and can be classified as glyphosate resistant or sensitive [31].

Here, we investigated how aquatic bacteria evolve in a semi-natural community faced with the GBH Roundup as an experimentally applied selective pressure. We exposed replicate mesocosms to one pulse of Roundup and sequenced metagenomes at two time points over 4 weeks. We sought to identify common genetic targets of GBH-driven selection. We first confirm that GBH affects overall community composition, then analyze diversity within species. We show that GBH often selects for fixed nucleotide substitutions without affecting overall within-species diversity. GBH consistently selects for nonsynonymous single-nucleotide variants in genes involved in amino acid transport and metabolism, including *aroA,* encoding the target enzyme of GBH. Overall, we demonstrate ecological and evolutionary changes occurring simultaneously in response to a known selective pressure over a timescale of 1 month.

## Materials and methods

### Experimental design, sampling, and sequencing

In this experiment, nine “ponds” (mesocosms) were filled with ~1000 L of water originating from Lac Hertel, a pristine lake with no known prior herbicide or pesticide contamination, at the Large Experimental Array of Ponds (LEAP) in the Gault Nature Reserve in Mont-Saint-Hilaire, Quebec. The ponds were filled throughout the week of 21 June 2021 and allowed to equilibrate for ~3 weeks before beginning the experiment. We sampled the nine ponds on 16 July 2021 (Day 0) and 13 August 2021 (Day 28). Four out of nine ponds received a 15 mg/L pulse of GBH (Roundup Super Concentrate Grass and Weed Control reg. no. 22759; Bayer) on Day 0 after the first sample was taken. One control pond received a 320 μg/L pulse of phosphorus (K_2_PO_4_). The day 0 sample from this pond was included in the co-assembly (described below) but was otherwise excluded from downstream analyses due to lack of replication. The four remaining control ponds did not receive any treatment. At both sampling points, we collected 1 L of water from each pond and filtered 250 ml through a 0.22 μm pore-size polyethersulfone membrane (Sigma-Aldrich) to collect the bacterial community for metagenomic sequencing. The filters were stored at −80**°**C prior to DNA extraction. DNA was extracted using the DNeasy PowerWater Kit (QIAGEN); libraries were prepared with the NEBNext Ultra II DNA Library Prep kit (New England Biolabs) and sequenced on an Illumina NovaSeqX with 150 bp paired-end reads.

### Metagenomic assembly, binning, and classification of metagenome-assembled genomes

Metagenomic reads were trimmed with trimmomatic v0.39 [32] to remove Illumina adapters and discard low quality reads. Next, we co-assembled metagenomic reads from samples collected on Day 0 (before any pond received GBH) with MEGAHIT v1.2.9 [33, 34]. We mapped reads from Day 0 using bowtie2 v2.5.4 [35] to the co-assembly and used metabat2 [36] to bin contigs into 324 bins. We then used anvi’o v8 [37] to generate a contig database from the Day 0 co-assembly, identify genes with Prodigal v2.6.3 [38], and annotate the database with single-copy gene taxonomy with HMMER [39] and profile contigs longer than 2500 bp by calculating coverage and single-nucleotide variants across samples. Bins with <50% completion were discarded. Bins with >50% completion were manually refined using the anvi’o interactive interface [37] following the guidelines in “Refining a bin using anvi’o” [40]. Two hundred fifty-nine bins that had >50% completion and <10% redundancy were considered metagenome-assembled genomes (MAGs). We used dRep [41] to confirm no two MAGs shared ≥95% average nucleotide identity (ANI), a commonly used species boundary [42]. Taxonomic classification of MAGs was done using the Genome Taxonomy Database (GTDB) Release 226 [43] with GTDB-Tk v2.4.0 [44, 45]. Functional annotation of genes was done using eggNOG mapper v2.1.13 [46, 47] and database v5.0.2 using DIAMOND [48] with default settings (e-value <0.001). MAGs were also annotated with Bakta [49] to extract *aroA* sequences that we classified as class I (GBH-sensitive) or class II (GBH-resistant) based on specific amino acid markers within the gene using the online classifier “*EPSPSClass* server” [31].

### Single-nucleotide variant calling

Metagenomic reads from both timepoints were competitively mapped to our MAG database using bowtie2 v2.5.4 [35]. We used inStrain v1.9.0 [50] to identify single-nucleotide variants (SNVs) in each MAG population at both time points with a minimum position coverage of 5× to call an SNV. We proceeded only with MAGs present at a minimum coverage of 5× and breath of 70% (Table S1). We discarded SNVs within 100 bp of contig edges and SNVs with a position depth greater than three times the MAG mean coverage or less than one-third of the MAG mean coverage. These depth filters remove both low- and high-coverage regions containing likely mismapped reads, using established cutoffs [8, 51]. The inStrain command “IS.get(‘covT’)” was used to extract the depth at each position to differentiate between positions where the reference frequency was 1 (and thus not reported in the output) and positions with a depth too low to call an SNV (<5×). We used inStrain to calculate the population ANI (which is based on the nucleotide identity of the reads compared to the reference MAG) of each population in each pond.

There was no evident association between the number of SNV calls (polymorphisms per Mbp) and MAG coverage (Fig. S1A). Nevertheless, to control for any subtle coverage bias in SNV detection, we subsampled mapped reads with samtools v1.21 [52] so each MAG had an average depth of coverage of 5×. While subsampling did reduce the number of SNVs called, the number of raw and subsampled SNV calls were correlated (Fig. S1B). For key analyses, we report results for both the raw and subsampled SNV calls. We subsampled the reads five times independently to assess repeatability of the number of SNVs called. The standard deviation scaled with the mean SNV rate in a MAG (Fig. S2A). Across all MAGs and resamplings, the relative standard deviations (SD/mean SNVs) were higher in MAGs with fewer SNV calls (Fig. S2B). The MAG with the highest relative SD had a mean of 116 SNVs, a minimum of 93 SNVs, and maximum of 145 SNVs across resamplings. The variation in SNV calling was generally much smaller than this example so we consider the estimated SNV rates to be reliable.

### Community composition of ponds

Metagenomic reads from each pond were subsampled with samtools v1.21 [52] to match the pond with the lowest number of reads (87 942 340 paired reads). We used coverM v0.7.0. [53] to estimate the relative abundance of our 259 MAGs across samples. We considered a MAG present if it had a relative abundance of >0.01% and breadth >10%. The relative abundance cutoff is low because the percent of total reads mapping to the MAG database is low (Table S2). To classify the community with a read-based approach, we used Kraken2 v2.1.3 [54, 55] and Bracken v3.0.1 [56] with the Kraken standard database (version date 07/14/25). We calculated the alpha diversity (Shannon Index) and beta diversity (Bray–Curtis dissimilarity) with Kraken for each sample.

### Gene copy number variation

We estimated gene copy number as the gene’s depth of coverage divided by the MAG depth of coverage. Genes with a copy number > 3 (likely enriched in mismapped reads) in any pond were excluded from the analysis. For each gene, we calculated the average gene copy number for control and GBH ponds at Day 0 and Day 28. GBH gene losses were identified as genes that went from ≥0.6 copies on Day 0 to ≤0.1 copies on Day 28 in GBH ponds while remaining at ≥0.6 copies in controls. GBH gene gains were identified as genes that increased by ≥0.7 copies between Day 0 and 28 in GBH ponds and had no increase in copy number in controls.

### Identifying genes with shifts in single-nucleotide variant frequency between treatments

We identified genes with shifts in SNV frequency between GBH and control ponds through time. We restricted this analysis to genes with a copy number around 1 (0.6–1.2 copies) to avoid SNV frequency changes being confounded by gene gain and loss events. For each SNV, we calculated the mean reference allele frequency in control ponds and in GBH ponds on Day 0 and Day 28. We identified SNVs that diverged in frequency between GBH ponds and control ponds through time. These were SNVs that started with an average reference allele frequency within ≤0.3 between GBH and control on Day 0 and then diverged by ≥0.7 between GBH and control on Day 28. The difference in reference allele frequency in GBH ponds between Day 0 and Day 28 was required to be ≥0.7. We classified SNVs as nonsynonymous or synonymous using inStrain and determined which genes contain at least one SNV with a “significant” shift. As a sensitivity analysis, we also considered frequency divergences of 0.5 and 0.6, which yielded similar results as the 0.7 threshold.

### Statistical analyses

All statistical analyses and visualizations were performed in R v4.3.3 with ggplot2 [57, 58]. We used vegan v2.6–8 [59] to perform PERMANOVAs using 1000 permutations on Bray–Curtis dissimilarity matrices at Day 0 and Day 28 calculated by Kraken at the level of phylum, class, genus, and species. We performed principal coordinate analysis (PCoA) on the Bray–Curtis dissimilarity with ape v5.8.1 [60]. All generalized linear mixed models (GLMMs) were performed using the glmer() function in lme4 v1.1–37 [61] assuming a Poisson distribution. Generalized linear models (GLMs) were performed using the glm() function in stats (R v4.3.3). To identify gene functions consistently under selection across MAGs, we performed a gene set enrichment analysis using the Clusters of Orthologous Genes (COG) 2020 database [62]. Contingency tables were created for each COG category from our lists of significant genes (genes with allele frequency shifts and gene losses) and a background set of all genes from the seven MAGs included in the analysis. We performed a Fisher’s exact test on each contingency table to identify enriched or depleted COG categories. A false discovery rate (FDR) correction was applied to account for multiple comparisons using a threshold of 0.05.

## Results

### Glyphosate-based herbicide alters bacterioplankton community composition

In this experiment, we sampled 1000 L ponds (mesocosms) filled with pristine lake water to study how bacterioplankton evolve over 1 month after exposure to Roundup, a commonly used GBH. Before beginning the experiment, ponds were left to equilibrate for ~3 weeks. Treated ponds (*n* = 4) received one pulse of GBH at 15 mg/L, whereas controls (*n* = 4) received no treatment. After filtration and DNA extraction, we sequenced a median of 452 million metagenomic reads per sample (Table S2). We co-assembled reads from all ponds from the first timepoint (before any ponds were treated) and binned the resulting contigs into a database of 259 nonredundant MAGs, which we operationally define as distinct species using a 95% ANI threshold (Materials and Methods). These MAGs had an average size of 2.6 Mbp (SD 1.07), completeness of 81.8% (SD 13.69), and contamination of 2.5% (SD 2.14). A median of 87 million reads per sample were mapped to this MAG database (Table S2).

**Figure 1:**
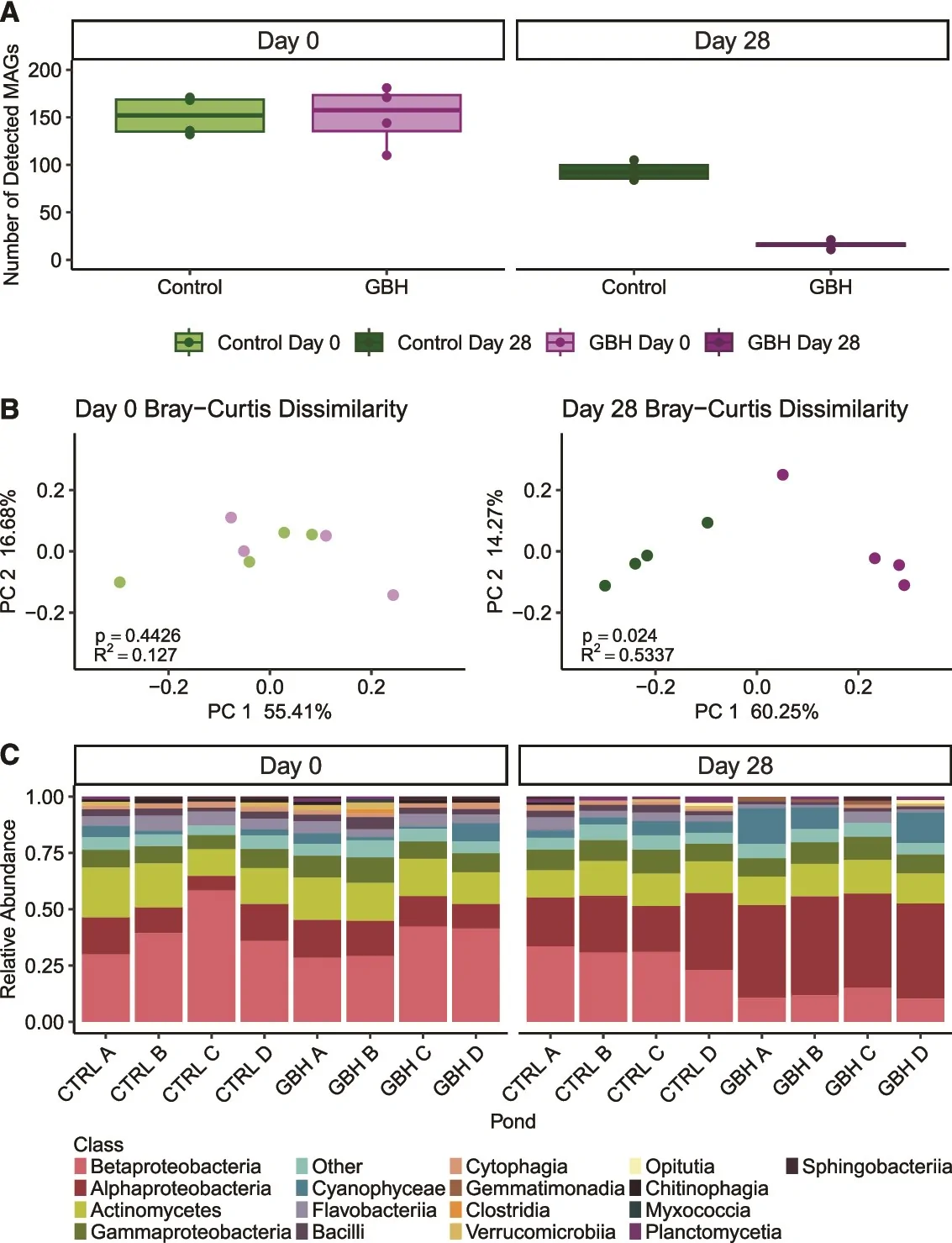
GBH treatment alters community structure. (A) Boxplots showing the number of MAGs present in control and GBH-treated ponds. A MAG was considered present if it was detected at 0.01% relative abundance with a minimum breadth of 0.1. The MAG database was constructed from metagenomic reads from all ponds on Day 0. Significance was assessed using a GLMM of MAG detection as a function of treatment, time, and their interaction (fixed effects) and pond as a random effect. *n* = 259 MAGs. (B) PCoA of Bray–Curtis dissimilarly between ponds at Day 0 and Day 28 calculated at the level of species inferred by mapping reads to the kraken2 database (Materials and Methods). *P*-values are from PERMANOVAs of Bray–Curtis dissimilarity matrices using 1000 permutations. (C) Barplots showing a GBH-driven shift in community composition. Taxonomic classification of metagenomic reads was done with kraken2. Classes with <1% relative abundance are grouped and displayed as “Other.”

Previous experiments in the same system of mesocosms showed that GBH treatment results in ecological changes in the bacterial community [2, 28]. If GBH inhibits the growth or viability of some species, we would expect MAGs present on Day 0 to be disproportionately lost in GBH-treated ponds compared to control ponds on Day 28. Consistent with this hypothesis, we found that GBH treatment significantly reduced the number of detected Day 0 MAGs over time. While a similar number of MAGs were detected in all ponds on Day 0 (range 110–181), at Day 28, ~10-fold fewer of these initial MAGs were detected in GBH ponds than controls (Fig. 1A; GLMM, effect of interaction between GBH treatment and time: *P* < 2E-16). This suggests that many MAGs in the initial pond community are susceptible to GBH. Time on its own was also a significant but weaker effect, with MAGs being lost over time in all ponds (Fig. 1A; GLMM, effect of time: *P* = 1.31E-13). This suggests a temporal succession effect common to all ponds.

Since our MAG database was constructed from only Day 0 samples and does not capture the full diversity of the ponds (particularly for taxa at lower abundance), we characterized the bacterial community in each pond and time point using a reads-based approach (Materials and Methods). If GBH alters the bacterial community, we would expect GBH ponds to contain distinct bacterial communities on Day 28 compared to control ponds. As expected, on Day 0, prior to any pond receiving GBH treatment, ponds did not cluster by treatment (Fig. 1B). On Day 28, GBH ponds had significantly different bacterial communities than control ponds at the level of species (Fig. 1B; PERMANOVA, *R*^2^ = 0.53, *P* = .024; Table S3). We additionally found significant shifts on Day 28 at the level of genus (*R*^2^ = 0.56, *P* = .020) and class (*R*^2^ = 0.62, *P* = .0280) but less so for phyla (*R*^2^ = 0.34, *P* = .070). The GBH-driven shift in community composition involved a switch from betaproteobacteria to alphaproteobacteria as the dominant taxonomic class (Fig. 1C). Despite the shift in community composition, we did not find a significant difference in alpha diversity (Shannon Index) between GBH and control ponds on Day 28 (Wilcoxon rank-sum, W = 10, *P* = .69). This indicates that the loss of taxa detectable as MAGs at Day 0 is compensated by the detection of new taxa at the end of the experiment, yielding no net change in alpha diversity.

### Quantifying within-species diversity across experimental treatments

**Figure 2:**
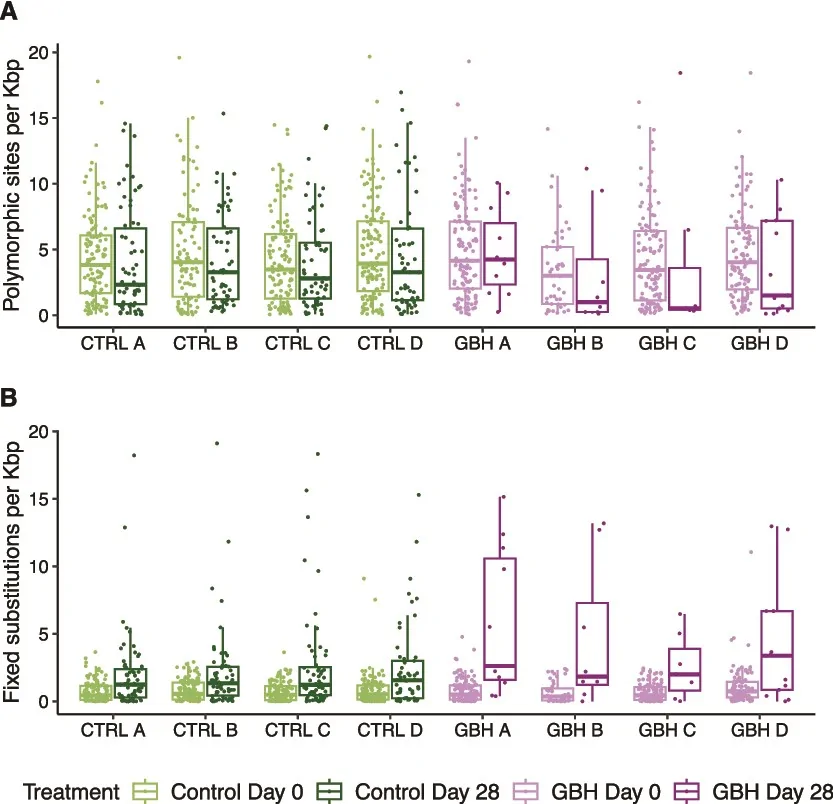
GBH treatment does not affect polymorphism but increases fixed substitutions within MAGs. The reference allele frequency at each genome position was calculated as the proportion of reads mapping to that position in the reference MAG that match the reference allele. Significance was assessed with GLMMs of polymorphic sites or fixed substitutions in a MAG as a function of treatment, time, and their interaction (fixed effects) and pond as a random effect. *n* = 232 unique MAGs, ranging from 7–132 MAGs per pond. (A) Total number of polymorphic sites (0 < reference allele frequency < 1) in the MAG population divided by the MAG genome size (bp) × 1000. (B) The total number of fixed substitutions (reference allele frequency = 0) in the MAG population divided by the MAG genome size (bp) ×1000. See Fig. S3 for a version of this analysis including only MAGs that were present in at least one GBH pond at Day 28.

We competitively mapped reads from both time points to our nonredundant Day 0 MAG database and quantified the number and frequency of SNVs (polymorphic sites) in each MAG present at a minimum of 5× coverage and 70% breadth (Table S1) in each experimental pond. Since SNV detection can be correlated with coverage, we subsampled reads such that each MAG had a coverage of 5× in each pond. If GBH purges genetic diversity through genome-wide selective sweeps, we would expect MAGs in GBH ponds to have lower diversity than control ponds. Using a GLMM, we found that GBH treatment through time did not have a significant effect on polymorphism within MAGs (Fig. 2A; GLMM, effect of interaction between GBH treatment and time: *P* = .40), suggesting that genome-wide sweeps are rare or incomplete over the time scale of the experiment. Time, regardless of treatment, had a negative effect on polymorphism (Fig. 2A; GLMM, effect of time: *P* = .042), suggesting some nonselective loss of diversity over time.

We next asked if GBH-treated ponds had higher numbers of fixed substitutions resulting from selection of alternate alleles, compared to the consensus nucleotide in the reference MAG. We calculated the number of fixed SNVs (reference allele frequency = 0) per kbp in each MAG. We found that time had the largest significant effect on the number of substitutions (Fig. 2B; GLMM, effect of time: *P* < 2E-16), suggesting adaptation to the pond environment or drift over time. GBH treatment had a significant interactive effect with time indicating that GBH treatment further selects for fixations (Fig. 2B; GLMM, effect of interaction between GBH treatment and time: *P* = 1.09E-06). Restricting the analysis to only MAGs that were present in at least one GBH pond at Day 28, we observe the same increase in fixed substitutions without a reduction in polymorphism after GBH treatment (Fig. S3), indicating that this phenomenon is not driven by the loss of MAGs present at Day 0 that were no longer detected at Day 28. The trend of increased numbers of fixed SNVs over time in GBH-treated ponds relative to control ponds is discernible in most of seven well-covered MAGs, while none of these MAGs experience a consistent loss of polymorphic SNVs over time in GBH ponds relative to controls (Fig. S4). As detailed further below, some MAGs contain diversity close to the 95% ANI threshold and likely encompass multiple distinct strains or species, while other MAGs closer to 99% ANI likely represent a single species. MAGs are therefore a convenient but imperfect proxy for species. Together, these results show that GBH tends to drive the fixation of alternative alleles within MAGs without purging polymorphism.

### Eco-evolutionary coupling

A recent study of natural tree-hole bacterial communities found that the change in nonsynonymous SNV frequency within a MAG was positively correlated with changes in MAG abundance, suggesting a coupling between ecological and evolutionary changes [24]. This association was not driven by any known selective pressure, so it could be plausibly explained by drift. To quantify the degree of eco-evolutionary coupling in our data, we first identified the frequency of nonsynonymous variants (polymorphic sites and fixed substitutions) in each MAG at a minimum of 5× coverage in a pond on Day 0 and Day 28. Next, we fit two GLMs (separating MAGs from control and GBH-treated ponds) on the median change in frequency of nonsynonymous variants over time in a MAG with the change in MAG abundance as a fixed effect. By considering the median frequency change for each MAG rather than each SNV independently, we implicitly assume linkage of SNVs within a MAG, which may not be strictly true but is statistically conservative. In contrast with the tree-hole study [24], we found a tendency for MAGs that decrease in relative abundance after GBH treatment to experience larger nonsynonymous SNV frequency changes (GLM, estimate = −0.603, *P* = .044; Table S4; Fig. S5A). Taking a decrease in relative abundance as a proxy for the strength of GBH-driven selection, this result is consistent with larger changes in SNV frequency being driven by stronger selection. Although based on a relatively small sample of 10 sufficiently covered MAGs from GBH-treated ponds (of which two MAGs occur in two replicate ponds), we tentatively conclude that there is some degree of eco-evolutionary coupling in our experiment. In support of this coupling being driven by GBH-driven selection rather than drift, we observed no such coupling in control ponds (GLM, *P* = .374; Table S5; Fig. S5B).

### Identifying genetic targets of selection

Our replicated study design provides the opportunity to identify consistent genetic targets of GBH selection. To do so, we considered seven MAGs that were present at a minimum of 5× average depth of coverage in at least one control and one GBH pond before and after the GBH pulse (Table 1). Notably, four of these MAGs are Alphaproteobacteria, a class that rose to higher abundance after GBH treatment (Fig. 1C). With these seven MAGs, we used two different approaches to identify genes targeted by GBH selection: (i) those with large shifts in nonsynonymous SNV frequency between GBH and control ponds and (ii) genes that were lost in GBH ponds (Materials and Methods).

**Table 1:** Summary of the seven MAGs screened for targets of GBH selection. Taxonomy was determined with GTDB. Predicted sensitivity and resistance to glyphosate are indicated by (S) and (R), respectively. Population ANI is calculated by inStrain as: (breadth * length − positions with reference allele frequency = 0)/breadth * length. Note that there is overlap between the genes detected with synonymous or nonsynonymous SNV frequency changes, such that the total number of genes is less than the sum of the synonymous and nonsynonymous counts. Gene loss events by definition do not overlap with SNV frequency changes, since SNV frequency changes were not considered in genes with changes in copy number (Materials and Methods).

					Potential targets of selection		
					Number of genes detected with:		
Species	MAG ID	EPSPS Class	Population ANI range Day 0	Population ANI range Day 28	Synonymous SNV frequency changes	Nonsynonymous SNV frequency changes	GBH gene loss events
c__Bacteroidia;o__CAMBQF01;f__CAMBQF01;g__CAMBQF01;s__	MAG_00674_1	I (S)	0.961–0.999	0.999–0.999	1	0	0
c__Alphaproteobacteria;o__Sphingomonadales;f__Sphingomonadaceae;g__Sphingorhabdus_B;s__Sphingorhabdus_B sp021298455	MAG_00097_1	II (R)	0.999–0.999	0.971–0.998	2271	1822	50
c__Alphaproteobacteria;o__Sphingomonadales;f__Sphingomonadaceae;g__Erythrobacter;s__Erythrobacter sp027486035	MAG_00110_1	II (R)	0.999–0.999	0.998–0.999	1	0	0
c__Alphaproteobacteria;o__Sphingomonadales;f__Sphingomonadaceae;g__Sphingorhabdus_B;s__Sphingorhabdus_B sp027488785	MAG_00194_1	II (R)	0.999–0.999	0.959–0.999	1535	945	2
c__Verrucomicrobiia;o__Verrucomicrobiales;f__Verrucomicrobiaceae;g__Prosthecobacter;s__	MAG_00197_1	II (R)	0.999–0.999	0.997–0.999	83	60	12
c__Bacteroidia;o__Flavobacteriales;f__Sanyastnellaceae;g__JAIXWP01;s__JAIXWP01 sp027491075	MAG_00179_1	Unclassified	0.999–0.999	0.999–0.999	0	0	0
c__Alphaproteobacteria;o__Caulobacterales;f__Caulobacteraceae;g__Brevundimonas;s__Brevundimonas sp027487935	MAG_00201_1	Unclassified	0.999–0.999	0.971–0.999	2204	1904	106

In the first approach, we calculated the difference between the mean reference allele frequency at each nucleotide position in control ponds and GBH ponds on Day 0 and Day 28. We identified genes containing nucleotide positions where the average frequency decreased by ≥0.7 in GBH ponds but did not decrease substantially (<0.3) in the controls (Materials and Methods). We found that four out of seven populations had genes containing at least one nonsynonymous SNV frequency shift and six out of seven had genes with at least one synonymous frequency shift (Table 1; Tables S6 and S7).

Both relatively diverse (population ANI of 95.9 or more) and less diverse (population ANI of 99.7 or more) MAGs contained genes with SNV frequency changes between GBH and controls (Table 1). Notably, three relatively diverse MAGs (*Sphingorhabdus_B sp021298455*, *Sphingorhabdus_B sp027488785*, and *Brevundimonas sp027487935*), each potentially containing multiple distinct strains or species, each contained >1000 genes with SNV frequency changes, spanning a large fraction of the genome. These three MAGs each contained little population diversity (ANI ~99.9) in each pond at Day 0, suggesting that rare lineages (undetected at Day 0) rose to higher frequency by Day 28, along with linked genes under selection, thereby decreasing the population ANI (Table 1). It is unlikely that all these genes are targeted directly by GBH selection. Rather, a few genes targeted by selection are likely linked to “hitchhiking” genes on a clonal genome background. Such linkage effects make it challenging to pinpoint targets of natural selection within an individual species [63]. However, if there are common functional categories of genes under selection across species, these should be detectable using enrichment tests.

**Figure 3:**
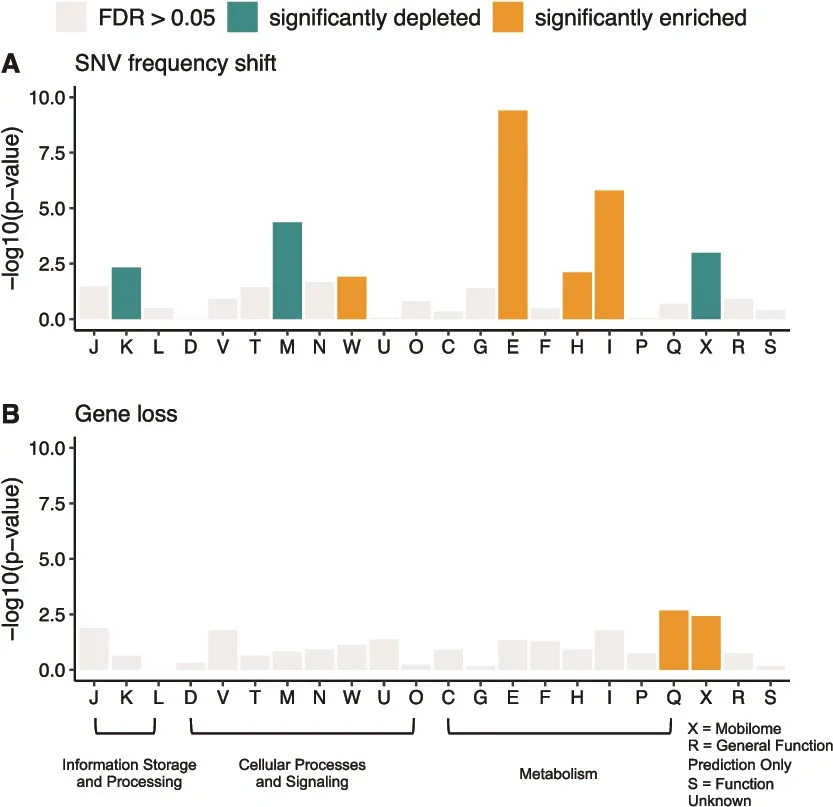
Functional categories enriched or depleted for genes containing GBH-driven changes in allele frequency (A) or gene loss (B). For each list of significant genes, a Fisher’s exact test was performed for each COG category between genes identified as targets of selection and all other genes present in the seven MAGs. Bars are colored by FDR-adjusted *P*-values and by odds ratio (<1 = depleted or >1 = enriched). Raw (unadjusted) *P*-values are shown on the *y*-axis. *P*-values that are not significant (FDR > 0.05) are shown as gray bars. COG categories: J = translation, ribosomal structure and biogenesis, K = transcription, L = replication, recombination, and repair, D = cell cycle control, cell division, chromosome partitioning, Y = nuclear structure, V = defense mechanisms, T = signal transduction mechanisms, M = cell wall/membrane/envelope biogenesis, N = cell motility, Z = cytoskeleton, W = extracellular structures, U = intracellular trafficking, secretion, and vesicular transport, O = posttranslational modification, protein turnover, chaperones, C = energy production and conversion, G = carbohydrate transport and metabolism, E = amino acid transport and metabolism, F = nucleotide transport and metabolism, H = coenzyme transport and metabolism, I = lipid transport and metabolism, P = inorganic ion transport and metabolism, Q = secondary metabolites biosynthesis, transport and catabolism, X = mobilome: prophages, transposons, R = general function prediction only, S = function unknown. See Fig. S6 for a version of this analysis including subsampled read data.

Across all MAGs, we found that genes with nonsynonymous SNV frequency changes between GBH and controls were significantly enriched in functional categories involved in metabolism (COG categories E, H, and I) and extracellular structures (W; Fisher’s exact test, *P* < .05, FDR < 0.05; Table S8; Fig. 3A). Among the genes in category E (amino acid metabolism) we found *aroA,* the known target of GBH, experiencing GBH-specific SNV frequency changes in MAGs *Sphingorhabdus_B sp021298455*, *Sphingorhabdus_B sp027488785*, and *Prosthecobacter sp.*, as well as other genes in the *aro* operon in MAGs *Sphingorhabdus_B sp021298455*, *Sphingorhabdus_B sp027488785*, and *Brevundimonas sp027487935* (Table 2). We additionally identified several operons that experienced similar numbers of nonsynonymous allele frequency shifts as the *aro* operon, such as *trp*, *cys*, and *met,* which are also involved in amino acid metabolism (Table S6). We found a significant depletion of nonsynonymous SNV frequency shifts in genes involved in transcription, cell wall/membrane/envelope biogenesis, and the mobilome (COG categories K, M, and X; Fisher’s exact test, *P* < .05, FDR < 0.05; Table S8; Fig. 3A). In a sensitivity analysis using lower reference allele frequency shifts of 0.5 or 0.6, we found similar functional enrichment profiles, particularly for metabolic genes (Tables S9–S12). These results point to specific gene functions consistently targeted by selection across populations. We repeated these analyses with the subsampled SNV data and obtained similar results, with GBH-driven SNV changes enriched in genes in COG categories E, I, and W and depleted in K (Fig. S6).

**Table 2:** Potential adaptive allele changes in the *aro* operon identified from the lists of GBH-driven allele frequency shifts in Tables S6 and S7.

Species	MAG ID	Gene	COG ID	Synonymous allele shifts	Nonsynonymous allele shifts
*Sphingorhabdus_B sp021298455*	MAG_00097_1	*aroA*	COG0128	33	13
*Sphingorhabdus_B sp027488785*	MAG_00194_1	*aroA*	COG0128	10	0
*Prosthecobacter sp.*	MAG_00197_1	*aroA*	COG0128	0	1
*Sphingorhabdus_B sp021298455*	MAG_00097_1	*aroB*	COG0337	26	5
*Sphingorhabdus_B sp027488785*	MAG_00194_1	*aroB*	COG0337	4	2
*Brevundimonas sp027487935*	MAG_00201_1	*aroB*	COG0337	32	17
*Sphingorhabdus_B sp021298455*	MAG_00097_1	*aroC*	COG0082	24	1
*Sphingorhabdus_B sp027488785*	MAG_00194_1	*aroC*	COG0082	6	0
*Brevundimonas sp027487935*	MAG_00201_1	*aroC*	COG0082	34	7
*Sphingorhabdus_B sp021298455*	MAG_00097_1	*aroE*	COG0169	21	3
*Sphingorhabdus_B sp027488785*	MAG_00194_1	*aroE*	COG0169	1	1
*Brevundimonas sp027487935*	MAG_00201_1	*aroE*	COG0169	27	10
*Sphingorhabdus_B sp021298455*	MAG_00097_1	*aroF*	COG3200	34	3
*Sphingorhabdus_B sp027488785*	MAG_00194_1	*aroF*	COG3200	1	0
*Sphingorhabdus_B sp021298455*	MAG_00097_1	*aroK*	COG0703	3	1
*Sphingorhabdus_B sp027488785*	MAG_00194_1	*aroK*	COG0703	2	3
*Brevundimonas sp027487935*	MAG_00201_1	*aroK*	COG0703	10	6
*Sphingorhabdus_B sp021298455*	MAG_00097_1	*aroQ*	COG0757	12	2
*Sphingorhabdus_B sp027488785*	MAG_00194_1	*aroQ*	COG0757	2	0
*Brevundimonas sp027487935*	MAG_00201_1	*aroQ*	COG0757	10	4

In a second set of selection tests, we identified genes that were lost in GBH-treated populations but not in control ponds. We estimated gene copy number by calculating the depth of coverage of a gene relative to the genome-wide average (Materials and Methods). We considered a gene lost if the copy number went from 0.6–1.2 to below 0.01 copies per genome. We found an enrichment for GBH-specific losses of genes involved in secondary metabolism and the mobilome (COG categories Q and X, Fisher’s exact test, *P* < .05, FDR < 0.05; Tables S13 and S14; Fig. 3B). Many of these gene losses were found in *Sphingorhabdus_B sp021298455* and *Brevundimonas sp027487935*, the two MAGs which experienced the most SNV frequency changes. We repeated this analysis with the subsampled data and found categories Q and X were below the level of significance (Fig. S6). We additionally looked for genes that were lost in controls but not in GBH ponds, as they could be advantageous in the presence of GBH, but no categories were significantly enriched (Tables S15 and S16). Similarly, no categories were enriched for GBH-specific gene gain events (Tables S17 and S18).

## Discussion

Our study is among a small but growing set of experimental studies exploring the fuzzy boundary between ecology and evolution in (semi) natural microbial communities [7, 24, 64, 65]. Previous work has shown that GBHs like Roundup alter the composition of aquatic bacterial communities [2, 28]. However, how individual populations within a community evolve in response to GBH is unknown. More generally, our understanding of microbial evolution in nature has been hindered by the lack of studies combining experimentally controlled selective pressures with measures of within-species diversity (evolution) in the context of a diverse community [66]. To fill this gap, we quantified diversity both among and within species in bacterioplankton experimentally exposed to GBH in freshwater mesocosm ponds.

Consistent with a previous experiment in the same mesocosm system [2], we show that GBH substantially restructures the bacterioplankton community without greatly impacting alpha diversity, reducing the number of MAGs that were present in the Day 0 community and allowing species not initially detected to predominate. While our conclusions regarding within-species evolutionary responses to GBH are limited to well-covered MAGs, it is possible that evolution is also occurring within rare species below our limit of metagenomic detection. Such rare species could be accessed by deeper metagenomic sequencing or more targeted culture-based approaches. Our short timescale of 28 days could be sufficient to detect changes in allele frequencies (including fixation) for fast-growing but perhaps not slow-growing species. Our conclusions therefore apply to the relatively abundant, fast-growing subset of populations accessible to relatively deep metagenomic sequencing (~200 M paired reads per sample).

Within the seven MAGs detected in both control and GBH-treated ponds at both time points, we identified genes potentially targeted by GBH-specific selection. These included those with large changes in SNV frequency or genes lost in GBH ponds but not controls. Three MAGs each contained thousands of genes with SNV frequency changes. Notably, each of these three MAGs contains population diversity as low as 95% ANI, close to the commonly used species boundary, suggesting substantial “strain”-level diversity within a MAG. It is implausible that each of these ~1000 genes are individually targeted by selection. More likely, GBH could have selected for a strain containing a few SNVs conferring GBH resistance, along with thousands of other hitchhiking SNVs. In isolation, it is statistically challenging to deconvolute SNVs conferring GBH resistance from hitchhikers. But viewed across MAGs, we can identify common targets of selection—at least at the level of broad functional categories. Using this across-MAG enrichment approach, we identified specific cellular pathways under consistent within-species selection in multiple species.

Glyphosate binds EPSPS (encoded by *aroA*) and prevents aromatic amino acid synthesis; we therefore expected selection on *aroA* and surrounding amino acid metabolic pathways. This expectation was largely met, along with some potential novel targets of selection. We classified each species as putatively resistant or sensitive to glyphosate at the beginning of the experiment based on known mutations in *aroA* [31]. Several MAGs with predicted GBH-resistant *aroA* alleles in their reference genomes still experienced GBH-specific SNV frequency changes in the *aro* operon. Why these putatively GBH-resistant MAGs would be targeted by GBH selection is not entirely clear, yet our results are consistent with a previous study showing EPSPS allele types to be relatively poor predictors of MAG survival in the face of extreme GBH stress [28]. A technical explanation is that the database matching EPSPS to putative resistance phenotypes is inaccurate or incompletely represents natural diversity. Another hypothesis is that GBH interferes with the cell in unknown ways, beyond the canonical EPSPS target. Selection on GBH-resistant species could also act indirectly. For example, GBH causes shifts in community composition, which could subsequently impose new selective pressures on these species through competition, cross feeding, or by creating novel niches [8, 15]. This could explain the enrichment of GBH-specific SNV changes in other metabolism genes, beyond those involved in amino acid metabolism. The enrichment of GBH-specific gene loss of mobile genes and secondary metabolism categories is intriguing, albeit difficult to interpret because the enrichment signal was not robust to downsampling reads. We nevertheless speculate that genes in these categories tend to be more dispensable under GBH selection and that selection to cope with GBH overwhelms any selection to retain these functions. Testing these hypotheses would require more targeted experimental study.

We report three key observations, with some important caveats in their interpretation. First, GBH is likely a strong selective pressure, as evidenced by the large shifts in community composition and within-MAG diversity. Our second key observation is that GBH does not purge within-species (MAG) genetic diversity (polymorphism) but does lead to an excess of fixed substitutions. This indicates that classic genome-wide selective sweeps, which are expected to purge within-species diversity genome-wide, are not measurably occurring on the time scales of our experiment. Yet genome-wide linkage is extensive in at least some MAGs, with thousands of genes experiencing changes in SNV frequency within a MAG, likely due to a shared genome background with the target(s) of selection. Our third observation is that we can statistically identify common gene functions targeted by selection within multiple MAGs, despite these linkage effects.

If genome-wide selective sweeps are rare, what could explain the maintenance of within-MAG polymorphism despite pervasive linkage, which should drag polymorphic SNVs to fixation along with the targets of selection? Could gene-specific selective sweeps explain our results? In the gene-sweep model, one or a few genes targeted by selection spread by recombination across genomic backgrounds, purging diversity locally in a few regions of the genome but leaving genome-wide polymorphism largely intact [19, 20]. Such a scenario could plausibly apply to the three MAGs with ~1–100 genes targeted by GBH selection but not to the three with >1000 genes under selection. A third plausible scenario involves “soft” selective sweeps, in which multiple lineages (strains) containing pre-existing GBH resistance–conferring alleles rise in frequency, eventually fixing on different lineage backgrounds, preserving ancestral polymorphism within the population. Confidently distinguishing among these scenarios would require quantifying genetic linkage, which is currently lacking in our metagenomic short-read data. Using whole-genome sequences from cultured isolates would allow the reconstruction of phylogenies to infer population (strain) structure and identification of recombination events potentially involved in gene-specific selective sweeps. Yet our metagenomic approach allowed us to track evolution relatively quickly and inexpensively across multiple species simultaneously, including those that might be difficult to culture.

For simplicity, we relied on a commonly used but arbitrary operational species definition at 95% ANI. At this threshold, each MAG is clearly a different species than other MAGs in the dataset, but it is possible that a MAG could encompass multiple ecological or biological species. Rather than arbitrary sequence identity cutoffs, ecological species are defined based on shared niche preferences and biological species by barriers to gene flow [17–20]. A majority of sequenced bacterial genomes appear to fit some semblance of biological species, with greater gene flow within than between species [67]. Species-like units defined by gene flow can be identified well above 95% ANI [68]. Therefore, metagenomic reads recruited to MAGs provide only a rough proxy for within-species diversity.

Finally, our 4-week time scale might have been too short to observe sweeps proceeding to completion. A potential genome-wide sweep took years to complete in a natural lake [22], much longer than the time scale of our experiment. However, we applied a strong selective pressure that could have accelerated the fixation of beneficial alleles, which we were able to observe by the end of the experiment. Further research will be needed to quantify selective sweep dynamics over longer time scales, in response to different selective pressures, and in a larger number of taxa.

## Supplementary Material

Web_Material_ycag201

## Data Availability

Metagenomic sequences from each sample are available on NCBI under BioProject PRJNA1346691.

## Code availability

Scripts for all analyses are available at https://github.com/emderrick/LEAP_sweeps.
