# Supplementary material for: Glyphosate-based herbicide exposure triggers genetic adaptation but not diversity collapse within bacterioplankton species
Source: ISME Commun. 2026 Aug 18;6(1):ycag201. doi: 10.1093/ismeco/ycag201 (PMC13615356; doi:10.1093/ismeco/ycag201)
Supplement: Supplementary file 1 [file web_material_ycag201.zip › Derrick_Supplementary_Information_revised.pdf]

Supplementary Figures and Tables for:

Glyphosate-based herbicide exposure triggers genetic adaptation but not diversity collapse within bacterioplankton species

Emma Derrick, Naíla Barbosa da Costa, Rowan D. H. Barrett, B. Jesse Shapiro

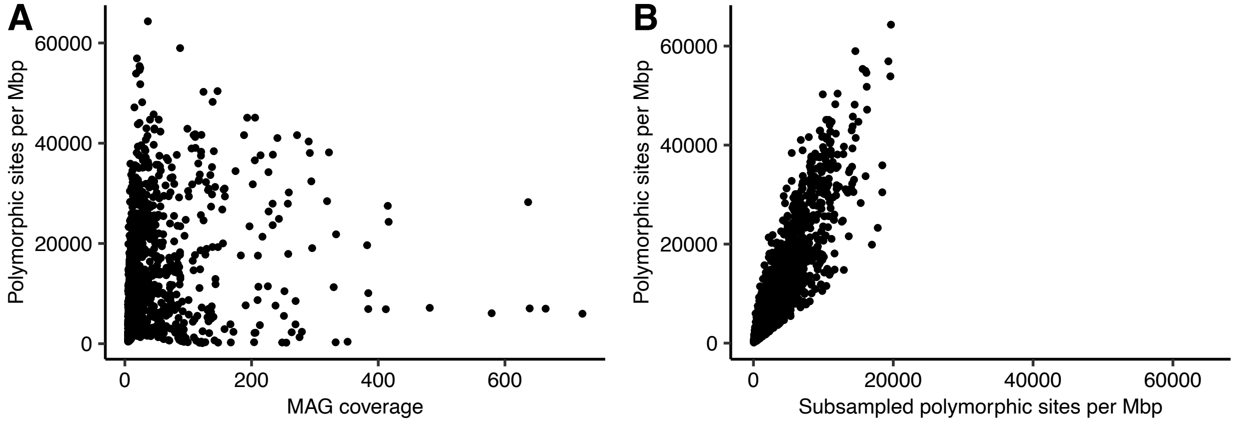

Supplementary Figure S1. Relationship between MAG coverage and read subsampling on SNV calls (polymorphic sites per Mbp per MAG). A) Relationship between the original MAG coverage and the number of polymorphic sites per Mbp. **B)** Relationship between the number of polymorphic sites before and after subsampling MAGs to 5x coverage.

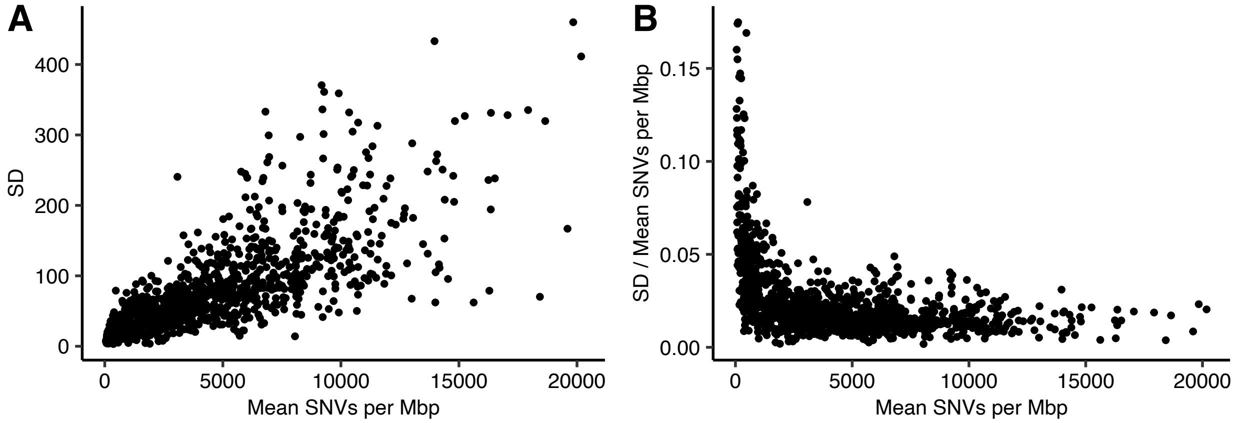

Supplementary Figure S2. Distribution of the number of SNV calls across subsamplings. **A)** Relationship between the mean SNVs per Mbp and the standard deviation between independent samplings. **B)** Relationship between the mean SNVs per Mbp of five subsamplings and the relative standard deviation. Populations with a low number of SNVs have a higher relative standard deviation.

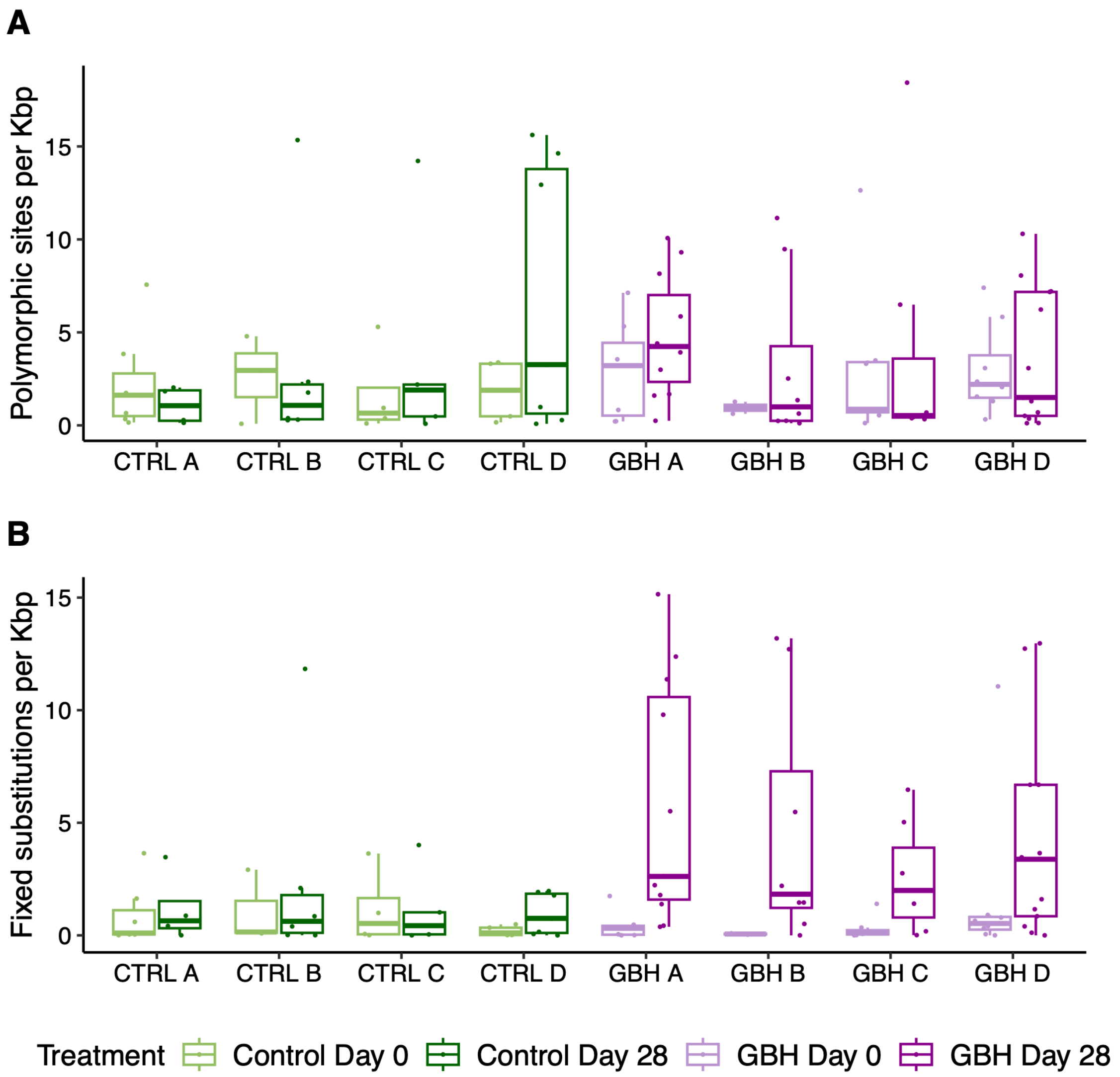

Supplementary Figure S3. Within-species diversity of a subset of MAGs using subsampled data. As a sensitivity analysis of the results presented in Figure 2, we restricted the figure to only include MAGs that were present in at least one GBH pond at day 28 (n = 17 unique MAGs, present in 2-12 ponds). The reference allele frequency at each genome position was calculated as the proportion of reads mapping to that site in the reference MAG that match the reference allele. **A)** Total number of polymorphic sites (0 < reference allele frequency < 1) in the MAG population divided by the MAG genome size (bp) x 1000. **B)** The total number of fixed substitutions (reference allele frequency = 0) in the MAG population divided by the MAG genome size (bp) x 1000.

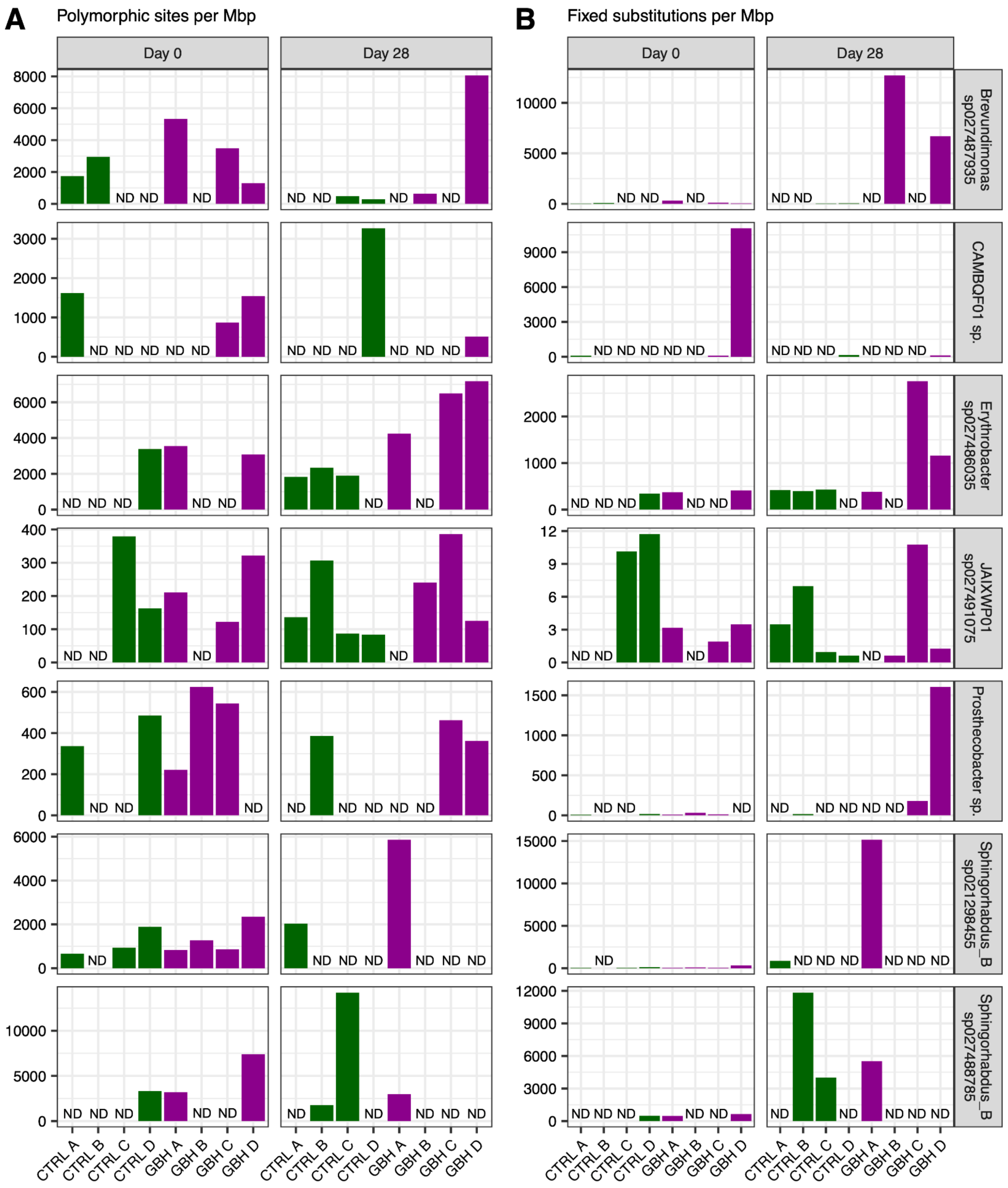

Supplementary Figure S4. Within-species diversity across time and treatments, shown for seven MAGs selected for identification of targets of selection using subsampled data. ND = MAG not detected (< 5x average coverage). **A)** Total number of polymorphic sites (0 < reference allele frequency < 1) from reads mapped to each MAG per Mbp. **B)** The total number of fixed substitutions (reference allele frequency = 0) per Mbp.

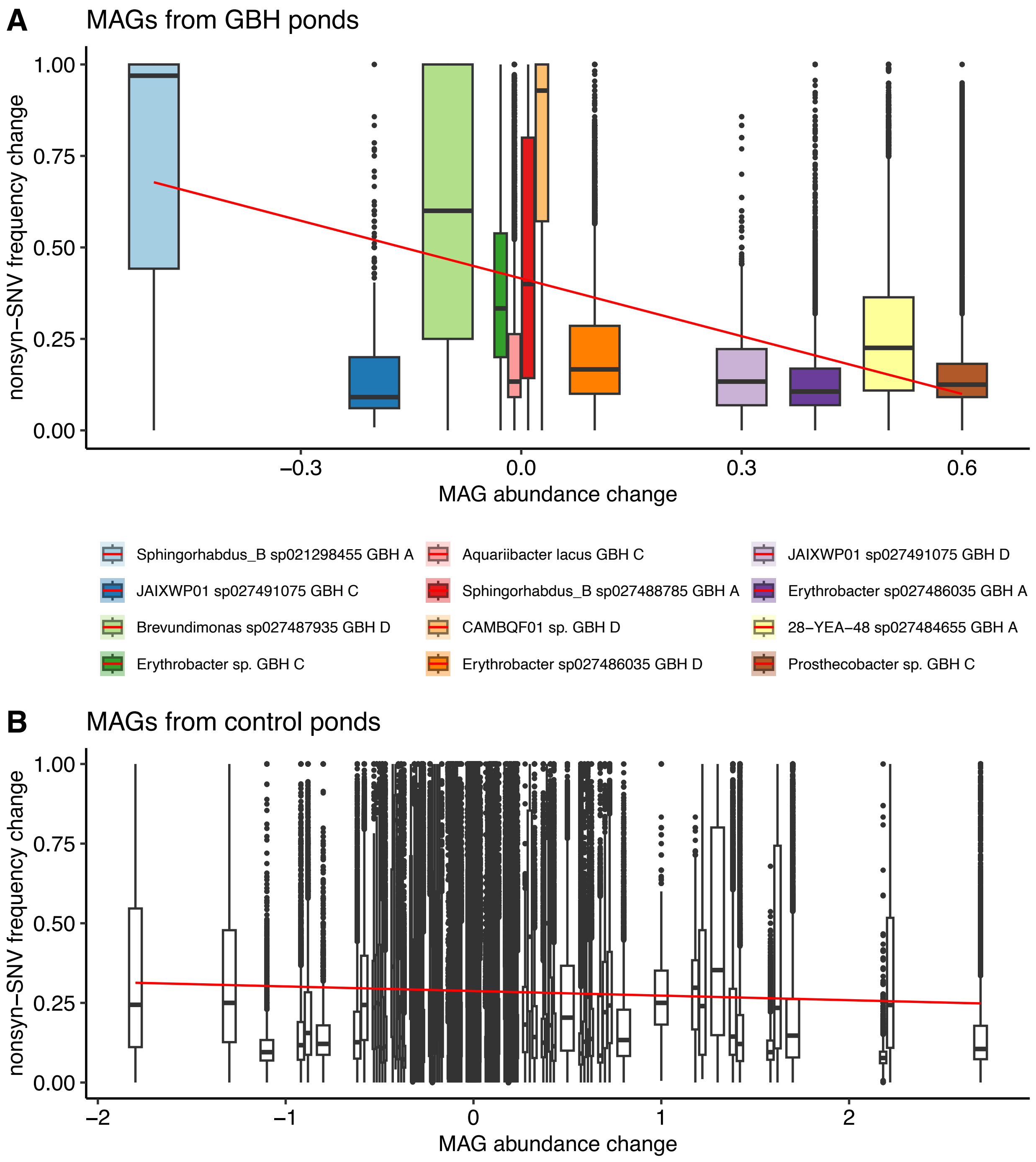

Supplementary Figure S5. The correlation between nonsynonymous SNV frequency and MAG abundance. Nonsynonymous SNV frequency change was calculated as the absolute value of the reference allele frequency on day 0 minus the reference allele frequency on day 28. MAG abundance change was calculated for each MAG by subtracting the MAG relative abundance on day 0 from the MAG relative abundance on day 28. A linear trendline was plotted considering all SNVs with method = “glm” in geom_smooth.

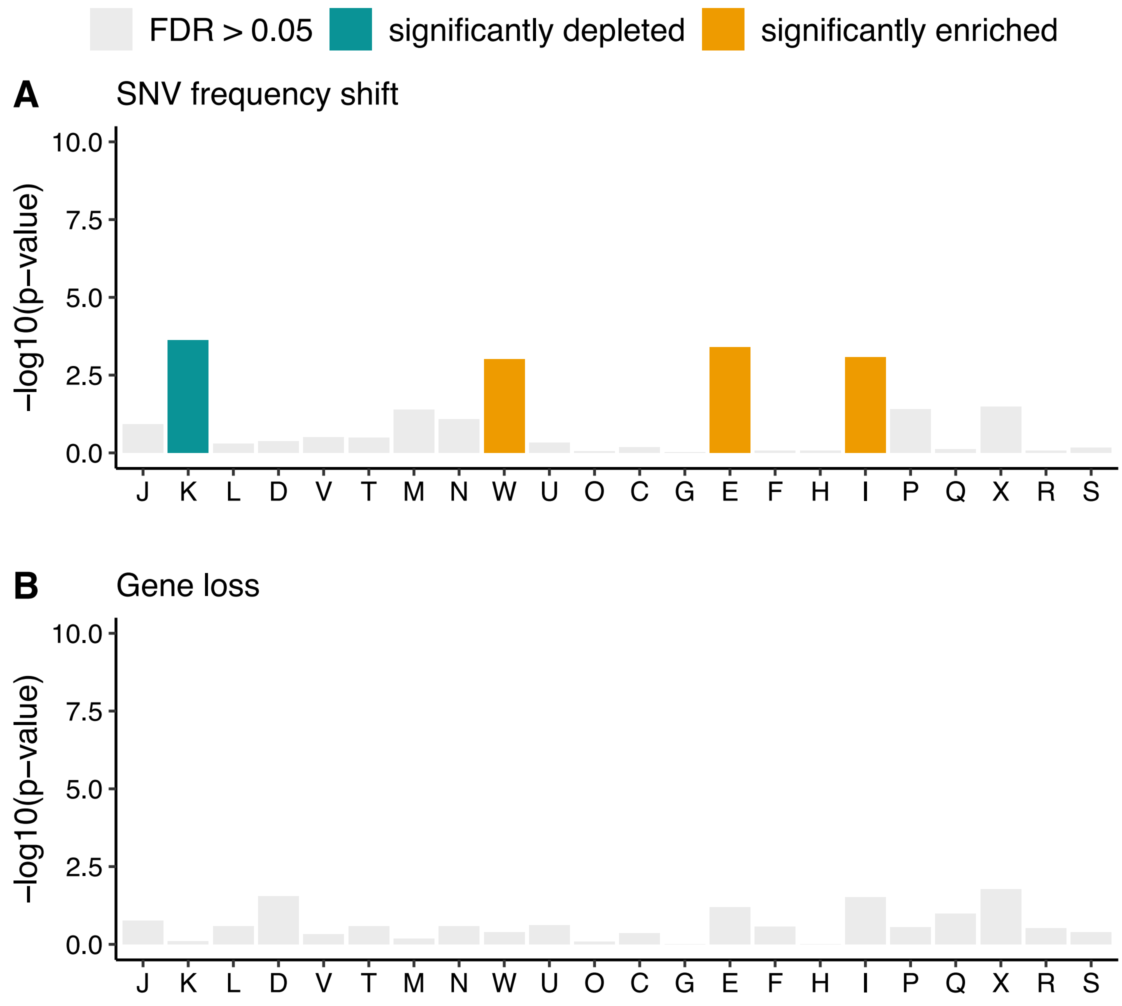

Supplementary Figure S6. Functional categories enriched or depleted for genes containing GBH-driven changes in allele frequency (A) or gene loss (B) using subsampled data. For each list of significant genes, a Fisher's exact test was performed for each COG category between genes identified as targets of selection and all other genes present in the seven MAGs. Bars are coloured by FDR adjusted P-values and by odds ratio (< 1 = depleted or > 1 = enriched). Raw (unadjusted) P-values are shown on the y-axis. P-values that are not significant (FDR > 0.05) are shown as gray bars. COG categories: J = Translation, ribosomal structure and biogenesis, K = Transcription, L = Replication, recombination, and repair, D = Cell cycle control, cell division, chromosome partitioning, Y = Nuclear structure, V = Defense mechanisms, T = Signal transduction mechanisms, M = Cell wall/membrane/envelope biogenesis, N = Cell motility, Z = Cytoskeleton, W = Extracellular structures, U = Intracellular trafficking, secretion, and vesicular transport, O = Posttranslational modification, protein turnover, chaperones, C = Energy production and conversion, G = Carbohydrate transport and metabolism, E = Amino acid transport and metabolism, F = Nucleotide transport and metabolism, H = Coenzyme transport and metabolism, I = Lipid transport and metabolism, P = Inorganic ion transport and metabolism, Q = Secondary metabolites biosynthesis, transport and catabolism, X = Mobilome: prophages, transposons, R = General function prediction only, S = Function unknown.

Supplementary Table S1. Coverage of MAGs present at a minimum of 5x coverage and 70% breadth across each pond. If blank the coverage is < 5x or the breadth is < 70%.

large table in .xlsx format

Supplementary Table S2. Summary of read count information and percent mapping to MAG database.

Sample	Pond	Timepoint	Total reads	Reads mapping to MAG database	% mapped
LEAP_META_01	CTRL A	Day 0	503846554	115665936	22.96
LEAP_META_02	CTRL B	Day 0	381336118	85543816	22.43
LEAP_META_03	CTRL E	Day 0	524163696	179812478	34.30
LEAP_META_04	CTRL C	Day 0	629272980	177512062	28.21
LEAP_META_05	CTRL D	Day 0	459603718	155646653	33.87
LEAP_META_06	GBH A	Day 0	444992726	128464812	28.87
LEAP_META_07	GBH B	Day 0	175884680	32473339	18.46
LEAP_META_08	GBH C	Day 0	459451618	119390368	25.99
LEAP_META_09	GBH D	Day 0	377423884	95470721	25.30
LEAP_META_10	CTRL A	Day 28	357365930	107134676	29.98
LEAP_META_11	CTRL B	Day 28	327770188	87220145	26.61
LEAP_META_12	CTRL E	Day 28	217334730	11610949	5.34
LEAP_META_13	CTRL C	Day 28	468856082	67797601	14.46
LEAP_META_14	CTRL D	Day 28	356206988	87128067	24.46
LEAP_META_15	GBH A	Day 28	469652644	17452479	3.72
LEAP_META_16	GBH B	Day 28	440153400	22449376	5.10
LEAP_META_17	GBH C	Day 28	856909050	34330044	4.01
LEAP_META_18	GBH D	Day 28	516681364	27621681	5.35

Supplementary Table S3. PERMANOVAs using 1000 permutations of beta-diversity (Bray-Curtis dissimilarity) between GBH-treated ponds and controls on day 0 and day 28. Beta diversity was calculated using Kraken 2 at the level of species, genus, class, and phylum. * $p < 0.05$.

Taxonomic Level	Factor	Time	R^2	*p*-value
Species	GBH	Day 0	0.127	0.4426
		Day 28	0.5337	0.0240*
Genus	GBH	Day 0	0.1292	0.4286
		Day 28	0.5574	0.0200*
Class	GBH	Day 0	0.1085	0.5405
		Day 28	0.6191	0.0280*
Phylum	GBH	Day 0	0.1246	0.4565
		Day 28	0.3374	0.0699

Supplementary Table S4. GLM for change in frequency of nonsynonymous variants within MAGs as the response variable and MAG abundance change as a fixed effect using only MAGs from GBH ponds. n = 10 unique MAGs. * $p < 0.05$.

Fixed Effects	Estimate	*p-value*
MAG abundance change	-0.60305	0.043886*

Supplementary Table S5. GLM for change in frequency of nonsynonymous variants within MAGs as the response variable and MAG abundance change as a fixed effect using only MAGs from control ponds. n = 84 unique MAGs. * $p < 0.05$.

Fixed Effects	Estimate	*p-value*
MAG abundance change	-0.01822	0.374

Supplementary Table S6. Summary of genes and their COG annotation that have a nonsynonymous shift in reference allele frequency in GBH ponds compared to controls using a cutoff of 0.7.

large table in .xlsx format

Supplementary Table S7. Summary of genes and their COG annotation that have a synonymous shift in reference allele frequency in GBH ponds compared to controls using a cutoff of 0.7.

large table in .xlsx format

Supplementary Table S8. Results of COG enrichment analysis using genes with a nonsynonymous SNV frequency shift using a cutoff of 0.7.

category	genes_num_category	genes_num_other	background_num_category	background_num_other	OR	p	fdr
E	440	3723	788	9921	1.48795183	4.05E-10	8.91E-09
I	393	3770	755	9954	1.37436436	1.60E-06	1.76E-05
M	306	3857	1012	9697	0.76020141	4.41E-05	0.00032339
X	15	4148	91	10618	0.42194305	0.00102292	0.00562606
K	198	3965	636	10073	0.7909039	0.00480047	0.02112207
H	284	3879	605	10104	1.22274677	0.00782119	0.0286777
W	45	4118	71	10638	1.63729829	0.01234747	0.03880634
N	80	4083	149	10560	1.38863548	0.02131187	0.05860763
J	321	3842	942	9767	0.86627934	0.03322397	0.07858382
T	199	3964	605	10104	0.83841182	0.03571992	0.07858382
G	183	3980	558	10151	0.83645513	0.03979862	0.07959725
R	402	3761	1126	9583	0.90967407	0.12506539	0.21164912
V	119	4044	361	10348	0.84349989	0.12099151	0.21164912
O	312	3851	730	9979	1.10750383	0.15235869	0.2394208
Q	127	4036	285	10424	1.15091197	0.20060904	0.2942266

F	128	4035	296	10413	1.11596504	0.32320325	0.41826302
L	221	3942	525	10184	1.08751371	0.31533776	0.41826302
S	242	3921	582	10127	1.07393093	0.37979684	0.46419614
C	266	3897	649	10060	1.05804591	0.44746964	0.51812275
P	249	3914	634	10075	1.01096082	0.87724296	0.91901644
U	109	4054	288	10421	0.97288087	0.86504344	0.91901644
D	76	4087	193	10516	1.01321643	0.9453728	0.9453728

Supplementary Table S9. Summary of genes and their COG annotation that have a nonsynonymous shift in reference allele frequency in GBH ponds compared to controls using a cutoff of 0.6.

large table in .xlsx format

Supplementary Table S10. Summary of genes and their COG annotation that have a nonsynonymous shift in reference allele frequency in GBH ponds compared to controls using a cutoff of 0.5.

large table in .xlsx format

Supplementary Table S11. Results of COG enrichment analysis using genes with a nonsynonymous SNV frequency shift using a cutoff of 0.6.

category	genes_num_category	genes_num_other	background_num_category	background_num_other	OR	p	fdr
E	460	3965	768	9679	1.462123	1.52E-09	3.35E-08
I	413	4012	735	9712	1.36022409	2.46E-06	2.70E-05
M	327	4098	991	9456	0.76139428	3.50E-05	0.00025671
X	16	4409	90	10357	0.41761044	0.0005887	0.00323786
H	308	4117	581	9866	1.27038345	0.0011358	0.00499753
K	211	4214	623	9824	0.78956562	0.00389049	0.01222725

T	203	4222	601	9846	0.78770421	0.00378324	0.01222725
G	190	4235	551	9896	0.80576477	0.01189772	0.03271872
W	46	4379	70	10377	1.55724399	0.02441426	0.05967931
N	82	4343	147	10300	1.32295147	0.04899096	0.10778012
J	347	4078	916	9531	0.88537091	0.0666613	0.13332261
C	293	4132	622	9825	1.12008113	0.12593892	0.23088802
R	430	3995	1098	9349	0.91646206	0.14788915	0.25027395
O	329	4096	713	9734	1.09657354	0.18202689	0.28604225
V	130	4295	350	10097	0.87318144	0.20463025	0.30012436
Q	133	4292	279	10168	1.12933623	0.25170369	0.34609258
L	234	4191	512	9935	1.08341815	0.32425232	0.39971311
S	258	4167	566	9881	1.08088795	0.327038	0.39971311
F	134	4291	290	10157	1.0937391	0.41887241	0.48501016
D	83	4342	186	10261	1.05454476	0.68672779	0.75540057
P	265	4160	618	9829	1.01314919	0.87935231	0.92122623
U	117	4308	280	10167	0.9861545	0.95563425	0.95563425

Supplementary Table S12. Results of COG enrichment analysis using genes with a nonsynonymous SNV frequency shift using a cutoff of 0.5.

category	genes_num_category	genes_num_other	background_num_category	background_num_other	OR	p	fdr
E	513	4648	715	8996	1.38865592	8.32E-08	1.83E-06
I	471	4690	677	9034	1.34010702	3.82E-06	4.20E-05
X	17	5144	89	9622	0.35729201	2.20E-05	0.00016155
M	396	4765	922	8789	0.79221092	0.00019118	0.00105147
J	392	4769	871	8840	0.83424354	0.00406008	0.01488696
T	241	4920	563	9148	0.79592052	0.00377438	0.01488696

K	253	4908	581	9130	0.81004774	0.00626508	0.01969024
C	351	4810	564	9147	1.18348189	0.01795111	0.04936555
H	339	4822	550	9161	1.17098865	0.02921127	0.07140532
W	50	5111	66	9645	1.42962594	0.06266299	0.13785858
N	91	5070	138	9573	1.24509476	0.10806901	0.21613801
L	274	4887	472	9239	1.0974663	0.23660171	0.43376981
O	378	4783	664	9047	1.07678235	0.2802256	0.47422795
G	244	4917	497	9214	0.91998646	0.30365201	0.47716745
P	317	4844	566	9145	1.05735885	0.44417381	0.61073898
V	158	5003	322	9389	0.92085246	0.43552977	0.61073898
Q	149	5012	263	9448	1.06797071	0.52914533	0.6847763
D	97	5064	172	9539	1.06231286	0.65113911	0.79583669
R	524	4637	1004	8707	0.98000665	0.73360739	0.84944014
F	150	5011	274	9437	1.03098002	0.79583541	0.87541895
S	288	4873	536	9175	1.01166648	0.88033438	0.92225507
U	137	5024	260	9451	0.99123209	0.95742508	0.95742508

Supplementary Table S13. Summary of genes and their COG annotation that are lost in GBH ponds.

large table in .xlsx format

Supplementary Table S14. Results of COG enrichment analysis using genes lost in GBH ponds.

category	genes_num_category	genes_num_other	background_num_category	background_num_other	OR	p	fdr
Q	10	112	407	14470	3.17435942	0.00214235	0.04165625
X	5	117	122	14755	5.16848816	0.00378693	0.04165625

I	17	105	1148	13729	1.93622864	0.01640148	0.07216652
J	3	119	1233	13644	0.27896706	0.01286708	0.07216652
V	9	113	470	14407	2.44140463	0.01626805	0.07216652
E	4	118	1235	13642	0.37444589	0.04573553	0.14374024
U	7	115	389	14488	2.26703923	0.04293109	0.14374024
F	0	122	427	14450	0	0.0523891	0.14407004
W	3	119	118	14759	3.15318331	0.07591507	0.18557017
C	3	119	912	13965	0.38602941	0.12403741	0.22740191
H	3	119	898	13979	0.39244072	0.12257104	0.22740191
N	4	118	227	14650	2.18771	0.1194546	0.22740191
M	6	116	1321	13556	0.53078911	0.14862305	0.25151593
P	11	111	887	13990	1.56301736	0.17475479	0.25867616
R	17	105	1518	13359	1.4248259	0.17637011	0.25867616
K	10	112	833	14044	1.50531641	0.23082796	0.29871854
T	3	119	808	14069	0.43896123	0.22220433	0.29871854
D	3	119	268	14609	1.37423178	0.4862816	0.59434417
O	10	112	1037	13840	1.19162419	0.590174	0.68335936
G	7	115	743	14134	1.15791445	0.67401391	0.72136311
S	5	117	823	14054	0.72976706	0.68857388	0.72136311
L	6	116	778	14099	0.93735041	1	1

Supplementary Table S15. Summary of genes and their COG annotation that are lost in control ponds.

large table in .xlsx format

Supplementary Table S16. Results of COG enrichment analysis using genes lost in control ponds.

category	genes_num_category	genes_num_other	background_num_category	background_num_other	OR	p	fdr
G	6	25	744	14224	4.5883871	0.00385682	0.08485002
Q	4	27	413	14555	5.22105641	0.01023161	0.11254766
K	3	28	840	14128	1.80204082	0.2518028	0.92327692
O	0	31	1047	13921	0	0.16731777	0.92327692
R	5	26	1530	13438	1.68903972	0.24110473	0.92327692
X	1	30	126	14842	3.92645503	0.23192419	0.92327692
E	4	27	1235	13733	1.64738342	0.31989958	0.94658179
L	0	31	784	14184	0	0.40804944	0.94658179
P	3	28	895	14073	1.68471668	0.43026445	0.94658179
S	0	31	828	14140	0	0.41505812	0.94658179
J	1	30	1235	13733	0.37066127	0.51243778	0.95147953
M	1	30	1326	13642	0.34293615	0.51898883	0.95147953
C	1	30	914	14054	0.51254559	1	1
D	0	31	271	14697	0	1	1
F	0	31	427	14541	0	1	1
H	1	30	900	14068	0.52103704	1	1
I	2	29	1163	13805	0.81863196	1	1
N	0	31	231	14737	0	1	1
T	2	29	809	14159	1.20702442	0.68360103	1
U	0	31	396	14572	0	1	1
V	0	31	479	14489	0	0.62570184	1
W	0	31	121	14847	0	1	1

Supplementary Table S17. Summary of genes and their COG annotation that are gained in GBH ponds

large table in .xlsx format

Supplementary Table S18. Results of COG enrichment analysis using genes gained in GBH ponds.

category	genes_num_category	genes_num_other	background_num_category	background_num_other	OR	p	fdr
V	9	82	470	14438	3.37161391	0.00247112	0.05436469
T	11	80	800	14108	2.4248125	0.01610204	0.17712248
I	2	89	1163	13745	0.2655859	0.04693489	0.34284116
O	11	80	1036	13872	1.84111969	0.06233476	0.34284116
F	0	91	427	14481	0	0.11692844	0.51448513
L	2	89	782	14126	0.4059312	0.24167321	0.8861351
G	2	89	748	14160	0.42540407	0.33000897	0.90752466
U	4	87	392	14516	1.70255688	0.30667552	0.90752466
D	0	91	271	14637	0	0.4162899	0.90838241
H	4	87	897	14011	0.71815374	0.66064175	0.90838241
K	6	85	837	14071	1.1866751	0.64499432	0.90838241
M	6	85	1321	13587	0.72602752	0.57855572	0.90838241
N	2	89	229	14679	1.44045925	0.65268166	0.90838241
P	7	84	891	14017	1.31098017	0.500865	0.90838241
R	7	84	1528	13380	0.72971204	0.60076987	0.90838241
X	1	90	126	14782	1.30352734	0.53982092	0.90838241
E	6	85	1233	13675	0.7828825	0.7033595	0.91022994
C	6	85	909	13999	1.08708989	0.82441452	1
J	7	84	1229	13679	0.9275156	1	1

category	genes_num_category	genes_num_other	background_num_category	background_num_other	OR	p	fdr
V	9	82	470	14438	3.37161391	0.00247112	0.05436469
Q	2	89	415	14493	0.78478408	1	1
S	5	86	823	14085	0.99501257	1	1
W	0	91	121	14787	0	1	1
